# Psychological experiences of parents of adolescent patients with non-suicidal self-injury: a qualitative study based on Bronfenbrenner’s ecological systems theory

**DOI:** 10.1186/s12888-025-06812-5

**Published:** 2025-04-11

**Authors:** Ya-Li Hu, Yue Shi, Gui-Zhi Qiao, Yan Sun

**Affiliations:** Department of Psychiatry, Shanxi Bethune Hospital, Shanxi Academy of Medical Sciences, Third Hospital of Shanxi Medical University, Tongji Shanxi Hospital, Taiyuan, 030032 Shanxi China

**Keywords:** Non-suicidal self-injury, Ecological systems theory, Parents, Psychological experiences, Qualitative study

## Abstract

**Background:**

Non-suicidal self-injury (NSSI)—an increasingly serious mental health issue among adolescents—is closely associated with several mental illnesses. Qualitative studies on parents of adolescents with NSSI behaviors in China—despite some advancements—have neglected to explore it as a result of individual–environment interactions. This qualitative study aimed to investigate the psychological experiences of parents of adolescent patients with NSSI based on Bronfenbrenner’s ecological systems theory, thus placing NSSI among adolescents in its corresponding social context.

**Methods:**

This qualitative study was conducted using face-to-face semistructured interviews between April and September 2024. The questions were set based on the Bronfenbrenner's ecological systems theory before the interview. Parents of adolescent inpatients with NSSI were recruited from the mental health department of a tertiary hospital in northern China. Colaizzi’s seven-step method was used to organize, analyze, and extract the themes from the data.

**Results:**

Eighteen parents were interviewed. The following four main themes emerged from the interview data: micro-system—coexistence of caregiver distress and growth (persistent negative emotions, positive experiences after self-adjustment, learning about mental illness, and improved disease management capabilities); meso-system—lifestyle changes (forced abandonment of social life, influence on other children, financial burden, and change in family atmosphere); exo-system—weak support system (limited resources of psychiatric services and desire for more supports); and macro-system—cultural environment.

**Conclusions:**

The social ecosystem of parents of adolescents with NSSI is undesirable. Psychological intervention, online and offline extension services, and the dissemination of professional knowledge may help improve the mental health level and care ability of parents of adolescents with NSSI, thereby reducing adolescent self-injurious behaviors.

**Supplementary Information:**

The online version contains supplementary material available at 10.1186/s12888-025-06812-5.

## Introduction

In recent years, non-suicidal self-injury (NSSI) among adolescents has become a significant public health concern, with increasing prevalence and profound implications for individuals, families, and society. NSSI refers to the deliberate and direct destruction of body tissue without suicidal intent, often manifested through behaviors such as cutting, scratching, burning, or biting oneself [1]. Research have indicated that NSSI is most prevalent during adolescence and early adulthood [2], with international studies reporting that approximately 22.9% of adolescents have engaged in NSSI at least once in their lifetime, and 18.6% have done so in the past year [3]. In China, the prevalence is even higher, reaching 26.9% [4]. Beyond physical harm, NSSI is associated with severe psychological consequences, including depression, anxiety, personality disorders, substance abuse, and an elevated risk of suicide [5, 6]. Given its far-reaching impact, understanding the experiences of parents of adolescents with NSSI is critical, as they play a pivotal role in their children’s recovery and well-being.

Existing research on NSSI has largely focused on individual-level factors, such as emotion regulation difficulties, which are central to many etiological models of NSSI [7–10]. Adolescents experiencing psychological distress often use NSSI as a maladaptive coping mechanism to regulate emotions, such as relieving irritability or numbing emotional pain [8]. However, NSSI is not solely an individual-level phenomenon; it is influenced by a complex interplay of environmental and interpersonal factors. For instance, Nock’s (2009) integrated model highlights the interaction between distal environmental factors (e.g., family environment) and proximal intrapersonal factors (e.g., emotion regulation) in the development and maintenance of NSSI [11]. Family dynamics, in particular, have been identified as a salient distal risk factor, with negative parenting behaviors—such as low parental warmth, excessive criticism, and neglect—being strongly associated with NSSI in adolescents [12–15]. Conversely, supportive family environments can mitigate the risk of NSSI and facilitate recovery [16]. Despite this recognition, the psychological experiences of parents and their role in the NSSI trajectory remain underexplored, particularly in the context of broader social and environmental influences.

Beyond the family, social factors such as peer relationships and school environments also play a critical role in adolescents with NSSI. Peer bullying, for example, has been identified as a significant risk factor, with victims of bullying being more likely to engage in self-injurious behaviors due to difficulties in emotion regulation, anxiety, and depression [17–19]. Schools that fail to foster a sense of belonging or fairness among students may further exacerbate these risks [18]. Parents, as primary caregivers, often find themselves navigating these complex social dynamics while trying to support their children, yet they frequently lack the knowledge and resources to do so effectively [20, 21]. This highlights the need for a more comprehensive framework that considers the interplay of individual, familial, and social factors in understanding NSSI and its impact on parents.

Bronfenbrenner’s ecological systems theory (EST) [22] provides a robust framework for addressing this gap. EST posits that human development is shaped by a series of nested and interacting systems. Micro-systems refer to individuals and include the physiological, psychological, and social factors that they possess, emphasizing individual needs, problems, and advantages. Meso-systems refer to groups and include family and social groups, predominantly referring to the influence of family and surrounding groups on individual development. Exo-systems are extensions of meso-systems, encompassing social structures that influence and define individuals, such as healthcare and education service systems. Finally, macro-systems pertain to the sociocultural environment wherein these three levels of systems exist [23]. This multi-level perspective is particularly relevant to NSSI, as it acknowledges the interplay between individual vulnerabilities and environmental contexts. By applying EST, this study moves beyond isolated factors to explore how interactions across micro-, meso-, exo-, and macro-systems shape the psychological experiences of parents of adolescents with NSSI. For example, the meso-systems focuses on parent–child interactions and family dynamics, the exo-system considers the role of external institutions (e.g., schools, healthcare systems) in influencing parental coping strategies, and the macro-system, in turn, examines how cultural attitudes toward mental health and NSSI may impact parents’ experiences and help-seeking behaviors.

This study represents a key conceptual breakthrough by integrating EST into the study of NSSI and parental experiences. While previous qualitative research has explored parents’ experiences in various settings [24–26], these studies have often overlooked the dynamic interactions between individual and environmental factors. By systematically categorizing parental experiences across the four levels of EST, this study provides a novel, holistic perspective that captures the complexity of NSSI and its impact on families. Furthermore, it highlights the added value of EST in advancing the understanding of NSSI by emphasizing the interconnectedness of individual behavior and the broader social environment. This approach not only deepens our understanding of the challenges faced by parents but also informs the development of multi-level interventions that address the diverse needs of families affected by NSSI.

In summary, this qualitative study aims to explore the psychological experiences of parents of adolescents with NSSI through the lens of Bronfenbrenner’s ecological systems theory. By examining the interplay of individual, familial, and social factors, the study seeks to identify common challenges and unmet needs among parents, providing a foundation for the development of targeted, family-focused interventions. The findings have the potential to inform mental health professionals, policymakers, and educators in designing more effective strategies to support families and reduce the prevalence of NSSI among adolescents.

## Methods

### Study design

We carried out a phenomenology qualitative study through face-to-face semi-structured interviews with parents of adolescents engaged in NSSI. Data were analyzed using Colaizzi’s seven-step method. The study conformed to the Consolidated criteria for Reporting Qualitative research [COREQ] checklist (see Additional file).

### Participants

Purposive sampling methods were used. We selected the parents of inpatient adolescents with NSSI in the Department of Mental Health of a tertiary hospital in Shanxi Province from April to September 2024. The inclusion criteria for adolescents were as follows: 1) engaged in NSSI behavior, and 2) aged 12 to 18 years. Based on the aforementioned criteria for adolescents, the inclusion criteria for parents were as follows: 1) were biological parents of adolescents, and 2) were able to complete the interview with normal intelligence and hearing and a junior high school education or above. The interviews were conducted iteratively, and we considered stopping recruiting more participants when there was no new themes or concepts emerged during the analysis of qualitative information. We screened adolescent patients who met the inclusion criteria through the electronic medical record system and subsequently scheduled appointments with adolescents’ parents who met inclusion criteria.

### Data collection

The research team developed a preliminary interview outline based on this study’s purpose, a literature review, and discussions with psychiatrists. Before commencing the interviews, three parents of adolescents who fulfilled the inclusion and exclusion criteria were selected for pre-interviews to further optimize the interview outline. Finally, we prepared an interview guide for each interviewee, listing the main questions and topics for the interview. The semi-structured interview guide was categorized into two sections (see Additional file). The first section (section 1) looked at the adolescent parents’ socio-demographic characteristics: gender, age, level of education, marital status, and the number of children. The second section (section 2) focused on parents’ psychological experiences on NSSI among adolescents based on Bronfenbrenner’s ecological systems theory.

Four authors (Yali Hu, Yue Shi, Guizhi Qiao, and Yan Sun) are female researchers, and they all have course training experience in qualitative research, including theoretical instruction and practical application. Sun Yan is the director of the psychiatric department, a registered psychotherapist of the Chinese Psychological Association, and has 30 years of medical experience in psychiatry. Semi-structured interviews were conducted by two researchers (Yali Hu and Yue Shi) who are licensed to practice mental health counseling, and both have extensive psychological nursing and communication skills. Yali Hu has a master's degree in medicine and has devoted herself to scientific research on non-suicidal self-injury in adolescents for 8 years. Yue Shi has a bachelor's degree in medicine and has been working in psychiatry for 13 years.

Prior to the interview, the respondents were informed of the purpose and significance of the study and promised to strictly follow the principle of confidentiality, utilizing codes instead of names to protect privacy. The interviews were conducted in the showroom of the Department of Mental Health, and the average duration of the interviews was 30–40 min. With the consent of the participants, the whole interview process was recorded in the form of audio recording. If the interviewee did not want to continue the interview for some reason in the middle of the interview, he/she could withdraw from the interview at any time.

### Data analysis

Phenomenological research data were derived from the interviewees’ direct, real-life experiences. This approach provides a unique philosophical framework for understanding human experience. By requiring researchers to immerse themselves in the *lifeworld*, bracket preconceptions, and prioritize description over interpretation, it ultimately reveals the universal meanings inherent in phenomena. Data collection and analysis were performed at the same time. Two researchers transcribed all the recordings verbatim and carefully checked the transcripts against the recordings to ensure accuracy. Further, data analysis was performed through NVivo12 software using the following seven-step phenomenological method elucidated by Colaizzi et al. [27]: 1. The data were carefully read and organized repeatedly to fully familiarize with and understand all the content provided by the participants. 2. Meaningful statements related to the research questions were extracted. 3. These statements were summarized, refined, and encoded. 4. The encoded statements were collated, and meaningful common concepts were identified to form a thematic cluster. 5. The thematic cluster was described in detail. 6. Similar themes and descriptions were compared, and similar views were extracted; thus, a final theme was formed. 7. Data analysis was conducted independently by 2 investigators. In case of disagreement, it was resolved through discussion at the study group meeting to ensure the reliability of the conclusion. The resulting thematic structure was returned to the interviewees for verification. Regarding peer debriefing, we have invited two psychiatrists specializing in qualitative research to review our study. Researchers endeavored to bracket their pre-existing knowledge and assumptions to avoid preconceived biases, achieving phenomenological bracketing (epoche) through maintaining reflective journals. Reflexivity was maintained throughout the research process.

### Ethics

Participants were given informed consent prior to the study. The interviewees were pseudonymized by assigning the alphabet P followed by a number in the order of recruitment (e.g., P1, P2, P3). All audio and text records are kept on a password-protected computer.

## Results

A total of 18 participants participated in this study, including 13 women (72.22%) and 5 men (27.78%), with a mean age of 47.06 ± 5.589 years. Table 1 presents the sample characteristics.

**Table 1:** Socio-demographic characteristics of participants

**Parents**						**Adolescents**		
**Participants (Parents)**	**Gender**	**Age (in years)**	**Education**	**Marital status**	**The number of children**	**Relationship with participants**	**Age (in years)**	**The method of NSSI**
P1	Female	45	College/University	Divorced	one	Daughter	18	Cutting
P2	Female	43	Less than high school	Married	two	Daughter	17	Poisoning
P3	Female	49	Less than high school	Married	two	Daughter	15	Cutting
P4	Male	63	Less than high school	Widowed	one	Daughter	17	Cutting
P5	Female	45	High school	Married	three	Son	16	Cutting
P6	Male	51	Less than high school	Married	one	Son	18	Cutting
P7	Female	49	Less than high school	Divorced	two	Daughter	15	Cutting
P8	Female	48	Postgraduate	Married	two	Son	16	Cutting
P9	Female	45	Less than high school	Divorced	one	Daughter	12	Cutting
P10	Male	45	College/University	Married	two	Daughter	16	Hitting
P11	Female	54	Senior high school	Married	two	Daughter	18	Poisoning
P12	Female	40	Junior high school	Married	two	Daughter	15	Cutting
P13	Female	45	Senior high school	Divorced	two	Daughter	12	Cutting
P14	Male	42	Less than high school	Married	one	Son	16	Cutting
P15	Female	46	College/University	Divorced	two	Daughter	13	Cutting
P16	Male	46	Less than high school	Married	one	Daughter	18	Cutting
P17	Female	52	Junior high school	Married	three	Son	16	Cutting
P18	Female	39	College/University	Divorced	one	Daughter	13	Cutting

**Fig. 1:**
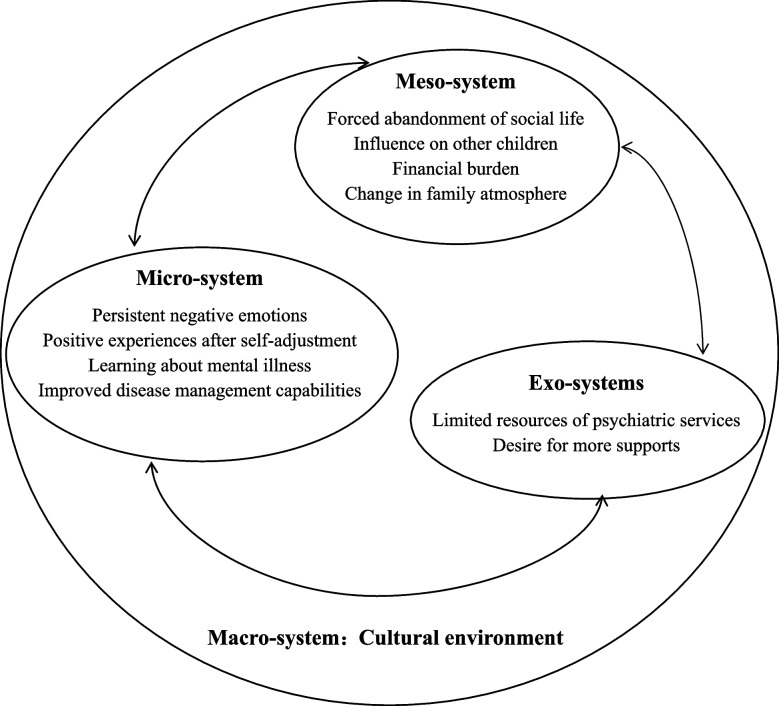
Themes and subthemes on the psychological experiences of parents of adolescent patients with NSSI

Based on EST, the psychological experiences of parents of adolescents with NSSI can be summarized into 4 themes and 10 sub-themes (Fig. 1).

### Theme 1: Micro-system -coexistence of caregiver distress and growth

#### Subtheme 1: Persistent negative emotions

##### Anxiety and fear

On seeing their adolescents’ scarred skin, participants experienced a series of negative emotions, such as worried, afraid, anxious, and sad.

“…Every day when I go home, I look in her bedroom and drawers to see if there are medicine boxes and knives. I worry and fear every day …” (P2)

“…I was worried all day. He went out and bought a knife to cut himself. If he had accidentally cut his blood vessels, he would have died …” (P6)

##### Guilt

Some participants attributed the NSSI behaviors to their lack of concern; some blamed themselves for not fulfilling their parental responsibilities by failing to detect these behaviors in time.

“…When I discovered my daughter's self-injurious behavior, I did not realize that she had psychological problems and did not provide timely help. If she had been treated earlier, she would not have ended up like this …” (P1).

“…When she was young, I only knew to ask her to help me in the restaurant. I did not care about her studies and frequently hit and scolded her … I neglected her growth …” (P7)

##### Sense of shame

All participants reported a clear sense of stigma, and to prevent their wards from being discriminated against by others, they kept their NSSI behaviors strictly confidential.

“…I did not tell any of my relatives, fearing that they would not understand and would say that my child had mental health issues …” (P3)

“…I did not mention to the school about my child's recent psychiatric hospitalization for the fear of my child being gossiped about by teachers and classmates …” (P8)

#### Subtheme 2: Positive experiences after self-adjustment

Most parents have scolded, neglected or failed to understand their children. Parents'attitudes may undergo some changes when they saw adolescents being diagnosed with emotional disorders or deliberately hurting themselves. Forgiveness, understanding, and support from parents could help adolescents reduce self-injury.

##### Parental companionship

Some parents believed that keeping their children company or talking to them when they were in a bad mood helped prevent NSSI behaviors.

“…I have been spending more time with her than before; especially after this hospitalization, I am with her 24 h a day. Her self-injury behaviors have significantly decreased …” (P9).

“…Her father was trying to spend as much time as possible with her, and her father's company gave her a great sense of security …” (P12).

“…After she was discharged from the hospital, I applied to the school for nonboarding. I will try spending more time with her in the future and will be the first to know when she has self-injurious thoughts …” (P13).

##### Parental understanding

Some parents made compromises and changes after learning about their children’s NSSI. They have been trying to accept their children’s thoughts and opinions.

“…I communicated more with my son and began valuing his ideas and opinions …” (P5).

“…I tried to understand and accept her negative emotions, so she began opening up and talking to me about her self-injury behavior …”(P18).

##### Lowering academic expectations

Some parents have chosen to lower their expectations of their children’s academic performance and hope that their children will grow up healthily rather than putting pressure on them.

“…I knew he was upset about his studies and hurt himself every time he failed his exams. Children’s physical and mental health is always the priority …”(P8).

“…Her mother is not well educated and is working in a supermarket. She hopes that our daughter will surpass her in all aspects and find a decent job in the future. When she learned that our daughter hurt herself, she lowered her expectations …”(P16).

#### Subtheme 3: Learning about mental illness

Some parents have started to learn about mental illnesses and how to get along with adolescents to prevent their children from hurting themselves again.

“…*I have started acquiring knowledge in psychology and accepting her current depressive state* …”*(P9).*

“…*I looked up depression online and realized that it was a disease requiring treatment, not merely relaxation* …”*(P14).*

“…*I specifically bought a book to learn about the characteristics of adolescent psychological development and how to interact with him, after which her self-injurious behavior has decreased* …”*(P15).*

#### Subtheme 4: Improved disease management capabilities

Some families have gained effective coping strategies through accumulated experience in dealing with their children's diseases, and their disease management capabilities have also been enhanced. Some parents are able to supervise effectively.

##### Removing dangerous objects

Some parents removed hazardous objects from their households to reduce NSSI occurrence among adolescents.

“…I asked my family members not to bring home any dangerous objects …”(P2).

“…The needles, scissors, and knives were locked up at home, but using her pocket money, she still bought some blades, which I would immediately confiscate as soon as I saw them …”(P11).

##### Medication and psychotherapy adherence

“…*After a week of medication and psychotherapy, she now has no NSSI-related thoughts. However, this is only the beginning, and I will supervise her to continue treatment after she is discharged* …”*(P4).*

### Theme 2: Meso-system -lifestyle changes

#### Subtheme 1: Forced abandonment of social life

Caring for adolescents forced some parents to give up their jobs and social circles. Owing to this long-term care burden, parents had limited time and energy for leisure and entertainment activities, which affected their social life.

“…*I quit my job and got a psychological counselor’s license myself. Now I mainly focus on treating my child’s disease. I do not care about anything else …”(P8).*

“…*Now all my thoughts are on my children, and I quit my job. I am not in the mood to dress up, and I usually do not have time to meet my friends …”(P18).*

#### Subtheme 2: Influence on other children

To avoid NSSI recurrence, some parents invested excessive efforts in caring for the affected children, precipitating psychological difficulties for their other children.

*“…She has already affected the second child, who is psychologically unbalanced. She says that we are too good to her sister and that everything is about her. She says, ‘Not only does my sister not have to go to school, she can also get anything she wants.’ The younger one does not want to go to school either and is also going for psychological counseling …”(P10).*

#### Subtheme 3: Financial burden

Some parents had to reduce their working hours or resign to take care of their children. Most parents reported that the financial burden was substantial.

“…Psychological counseling institutions are expensive, costing hundreds per hour. I am afraid our family cannot afford it for a long time …”(P3)

“…The doctor said that the medicine had to be taken for a long time, the cost of which is a large expense, and his father has been the sole breadwinner in the family …”(P17)

#### Subtheme 4: Change in family atmosphere

Parental relationships with—and attitudes toward—their wards play an important role in NSSI occurrence and reduction among adolescents. When parents create a relaxed and harmonious family atmosphere, adolescents’ emotional and behavioral problems change accordingly.

##### Parental relationship

“…Every time I argued with my wife, I displaced my bad feelings onto my daughter… When I first learned of her self-injurious behavior, I remembered her saying that she felt uncomfortable and depressed in front of us and that she would rather be alone. Later, I spoke with her mother about getting along amicably in the future and creating a harmonious family environment for our children …”(P10).

“…We quarrel frequently because of trivial issues. Her mother has a bad temper. Due to our son’s low mood and self-destructive behavior, we argue less than we did before and generally do not argue in front of him …”(P14).

##### Parent–child relationship

“…My daughter is actually terrified of her mother. When her mother came to the hospital yesterday, I observed that my daughter was nervous, shaking, and breathless. Her mother did not scold her as soon as they met like before, and she became slightly gentler …”(P16).

“…*My temper has become milder, and I communicate calmly with my son; he is willing to open up to me and tell me when he is having self-injurious thoughts …”(P17).*

### Theme 3: Exo-system -weak support system

#### Subtheme 1: Limited resources of psychiatric services

The lack of psychiatric services in some regions of our country precipitates difficulties in seeking medical treatment and increases caregivers’ care burden.

“…*Our county hospital does not have a psychiatric department. I consulted the psychology teacher at my child's school, but this had no effect, which is why I came to a Grade III A hospital like yours …”(P2).*

“…I hope that in the future, a psychiatric clinic is established in our hometown, so that we do not have to travel far for follow-up visits …”(P13)

#### Subtheme 2: Desire for more supports

##### Desire for hospital support

Most participants did not know how to avoid triggering children in their daily lives and wanted to learn communication skills for use with children during emergencies. Some participants hoped to receive professional guidance from medical staff to better care for adolescents and prevent NSSI occurrence while at home.

“…I hope that when my child experiences psychological problems at home, we can directly contact the doctor to help her through psychological counseling …”(P4)

“…I hope that the medical staff will teach us how to communicate with our children, such as parenting skills and daily communication …”(P11)

“…After going home, we want to ask the doctor through online consultation how to help the child stop the drug smoothly …”(P14)

“…I want to know that if my child suddenly develops a bad mood, how can I help her calm down …”(P15)

##### Desire for school support

Most parents expect assistance from school—including emotional support, acceptance, and respect—for their adolescent wards.

*“…As his teachers knew that my son engages in self-injurious behavior, we did not let him go to school fearing that he would commit suicide or NSSI at school, and we hoped that the school authorities could help us …”(P6).*

*“…I hope teachers also have basic psychological knowledge, timely discover and deal with adolescents'emotional problems, take better care of her, and allow her to take a leave …”(P12).*

### Theme 4: Macro-system -cultural environment

A macro-system describes the sociocultural environment wherein micro-, meso-, and exo-systems are embedded. Almost all parents, as well as relatives around them, lack an understanding of NSSI and exhibit a certain degree of cognitive misunderstanding vis-à-vis the disease. This precipitates an inability to recognize and respond effectively to NSSI in its early stages.

*“…At first, I could not understand that she had depression. For any bodily injury, pain, or inflammation, I would definitely take her to a doctor. Once, I accidentally found that her arm was scratched with a knife used for cutting colored paper. I thought nothing at that time …” (P1).*

“… I didn't know it was a disease. My relatives thought my daughter was in a rebellious stage …” (P4)

“…When I first discovered that she had scratched herself, I thought she was using an extreme way to relieve stress, but I did not expect it to be depression …”(P9).

## Discussion

The study findings demonstrated significant systemic dysfunction within caregivers'socioecological frameworks [22], particularly among parents of adolescents with NSSI behaviors. Caregivers bear prolonged multidimensional burdens, with challenges manifesting across micro-, meso-, exo-, and macro-systems. While the micro-system reveals caregivers’ psychosomatic exhaustion and ambivalent emotional states (e.g., guilt, shame), the meso- and exo-systems highlight familial, school, and healthcare access issues. Critically, medical staff often prioritize adolescent patients’ needs while neglecting parental well-being—a systemic gap requiring urgent attention.

### Micro-system: targeted psychological interventions for caregivers

At the micro-system level, parents’ persistent negative emotions (anxiety, guilt, shame) align with prior studies [28–30]. Some parents attribute NSSI behaviors to their own lack of care and experience a strong sense of guilt; meanwhile, some parents believe that their adolescent wards engaging in NSSI behaviors is shameful and are, thus, unwilling to tell others about it, reflecting parents’ helplessness and self-blame due to ineffective coping strategies. Therefore, group-based psychological therapy should be implemented among parents of adolescent patients with NSSI. Beyond its foundational principles [31], MBSR could be tailored for caregivers through 8-week group programs incorporating guided meditation, body scanning, and mindful communication exercises. For example, weekly sessions could focus on reducing parental guilt by reframing self-blame into self-compassion. PERMA Model: To operationalize Seligman’s framework [32], caregiver workshops could be designed to: (1) Cultivate positive emotions via gratitude journaling or strengths-based reflection. (2) Enhance engagement through collaborative problem-solving activities with peers. (3)Strengthen relationships via parent support groups fostering shared experiences. (4) Create meaning by helping parents reframe caregiving as a purposeful journey. (5) Celebrate accomplishments through milestone-based recognition (e.g., mastering a coping skill). Additionally, deep breathing, music therapy, or rational catharsis for stress relief could be integrated into individual counseling to help parents process guilt and shame [33]. These interventions should be delivered through hospital-based caregiver programs. By improving parental mental health, caregivers can better model emotional regulation for their adolescents, indirectly mitigating NSSI recurrence.

This study revealed that along with negative experiences, parents also gain positive experiences in the process of caring for adolescent patients with NSSI. For instance, family relations may become harmonious. The positive feelings and experiences that develop during the care process—that is, the sense of benefitting from the condition—help caregivers improve their ability to cope with negative events, thereby improving their physical and mental health [34]. Therefore, medical staff should adjust their perspective accordingly by focusing on and uncovering parents’ positive experiences during the care process—consistent with the recent positive psychological concept of post-traumatic growth [35].

### Meso-system: economic support and family-centered interventions

At the meso-system level, this study found that adolescent NSSI behaviors’ negative impact on families predominantly manifested as an economic burden and adverse effects on other children’s mental health outcomes and parents’ work—consistent with Townsend et al.’s findings [36]. Caring for adolescents with NSSI occurrences necessitates a significant amount of time, and some parents even quit or change jobs. To mitigate the care burden for parents of adolescent patients with NSSI, medical staff can provide online and offline extension services. Medical staff can fully integrate existing social, school, medical institutions, and other relevant resources; strengthen knowledge dissemination through news, websites, and other self-media platforms; and offer professional support to parents for a wide variety of problems. Simultaneously, for the period of adolescent patients’ stay at home, medical staff should use information technology to provide regular follow-up guidance, improve the cognitive level and coping ability of patients’ parents about NSSI, help parents take appropriate care of adolescents, and reduce unnecessary energy consumption. For patients from low-income families requiring multiple visits, follow-up intervals must be reasonably arranged to reduce their round-trip expenses or online diagnosis and treatment services may be offered to help reduce their financial burden.

Studies have underscored the pivotal role of family dynamics in mitigating NSSI among adolescents, with robust family functioning—specifically characterized by emotional intimacy and adaptive relational flexibility—demonstrating significant protective effects [37]. Therefore, it is crucial to develop family-centered interventions that engage family members and empower them to play an active role throughout the therapeutic process. In the future, the potential effectiveness of Family-Involved Acceptance and Commitment Therapy (FI-ACT) could be further explored among adolescent patients with NSSI. FI-ACT integrates the six core processes of traditional ACT (cognitive defusion, acceptance, present moment awareness, self-as-context, values, and committed action) with family systemic interventions, including weekly family sessions. Furthermore, Attachment-Based Family Therapy (ABFT) employs a series of process-oriented, emotion-focused techniques to address maladaptive behavioral patterns, strengthen family relationships—particularly parent-adolescent dynamics—and enhance overall family functioning, ultimately reducing adolescents’ engagement in NSSI [38]. Research also highlights that intergenerational transmission of risk factors and sibling bullying may lead to self-injurious behaviors [39, 40]. These findings underscore the necessity of expanding investigations to include extended family subsystems (e.g., grandparents, siblings) and developing multidimensional family-based intervention frameworks tailored to address these interconnected dynamics.

### Exo-system: cross-sector collaboration and parental support

At the exo-system level, our findings emphasized the urgent need for a coordinated healthcare-school-family framework to advance adolescent mental health literacy and address NSSI. The study identified significant gaps in parental awareness, particularly among mothers with limited education, who expressed a strong demand for specialized support—including training in adolescent communication and adaptive parenting strategies. Prior research highlights caregivers’ tendency to misinterpret early NSSI behaviors as typical teenage rebellion [26], often delaying professional intervention until self-injury escalates [41]. To bridge these gaps, healthcare providers should deliver targeted psychoeducation via multimedia platforms (e.g., micro-videos, infographics, pamphlets, and public health portals) to rectify cognitive misconceptions, improve parent–child dynamics, and foster responsive caregiving. Peer-led parent support networks could further facilitate experiential knowledge-sharing among families navigating NSSI challenges.

Concurrently, schools should collaborate with mental health professionals prioritize expert-led workshops on NSSI prevention, routine psychological screenings, tiered interventions for high-risk students (e.g., individualized counseling), and streamlined referrals to mental health institutions. Such systemic integration ensures early identification and continuity of support within educational settings.

### Macro-system: societal awareness and professional training

At the macro-system level, societal understanding of adolescent NSSI remained critically underdeveloped. Public health campaigns must prioritize disseminating scientific knowledge and expanding access to psychological services for affected families. Crucially, healthcare and educational systems require standardized training to address systemic shortcomings. The literature reported that healthcare professionals experience significant psychological stress when dealing with adolescent patients with NSSI [42, 43]. Existing literature revealed inconsistent preparedness among professionals: healthcare providers often lack standardized NSSI education and psychosocial training [44–46], while school staff report insufficient knowledge to recognize or address NSSI [47–49]. In addition, perceptions of friends'self-injurious behaviors were associated with adolescents'own self-injury [50]. Comprehensive training programs are urgently needed to equip these stakeholders with skills to support adolescents effectively.

## Limitations

While this study provides critical insights into the caregiving experiences of parents of adolescents with NSSI, several constraints warrant consideration.

Although thematic saturation was achieved with 18 participants, this study predominantly focused on maternal experiences, with limited paternal involvement, thereby constraining the generalizability of findings to the broader population of parents of adolescents with NSSI. The observed gender imbalance arises from two interconnected factors: (1) Hospital chaperone policies permitted only one parent to accompany adolescents during NSSI-related clinical consultations, with mothers overwhelmingly fulfilling this role. (2) Mothers are often designated as the primary caregiver, while fathers are positioned as financial providers rather than direct participants in emotional or medical care in Chinese culture. It is hoped that our future research will incorporate a more diverse sample of fathers, such as those from varying socioeconomic backgrounds and geographic locations. By employing a mixed-methods research approach to compare the differences in paternal and maternal subjective experiences of adolescent self-harm, we aim to further supplement the findings of this study.

The absence of assessment regarding the severity (e.g., frequency, methods, medical lethality) and duration of NSSI in the adolescents represented a critical limitation. These unmeasured variables are likely to influence caregivers’ emotional responses, coping strategies, and help-seeking behaviors. For instance, parents of adolescents with high-frequency NSSI may experience heightened helplessness, whereas those confronting medically risky behaviors (e.g., deep tissue cutting) might prioritize urgent clinical interventions over relational repair. By not accounting for these dimensions, the study risks homogenizing diverse parental experiences and overlooking nuanced patterns in how NSSI traits shape family dynamics.

## Conclusion

This qualitative study, grounded in EST, illuminates the multifaceted psychological experiences of parents caring for adolescents with NSSI. In the microsystem, parents navigated dual challenges of psychological distress and post-traumatic growth. The mesosystem analysis revealed parents’ experience lifestyle changes, and in the exosystem systemic gaps in healthcare and school support are emphasized. Most crucially, the macrosystem analysis uncovered a persistent societal underestimation of NSSI as a significant mental health concern. These findings suggested that family-centered interventions should be established to address caregiver mental health issues and psychoeducational programs should be developed to enhance the public's NSSI literacy and coping strategies, and the implementation of this ecological intervention framework has the potential to reduce the incidence of NSSI.

## Supplementary Information

Additional file 1. COREQ checklist.

Additional file 2. Semi-structured interview guide.

## Data Availability

Data are provided within the manuscript or the corresponding author Yan Sun, upon reasonable request.

## Abbreviations

NSSI: Non-Suicidal Self-Injury
BPD: Borderline Personality Disorder
BD: Bipolar Disorder
MDD: Major Depressive Disorder
COREQ: Consolidated criteria for reporting qualitative research
EST: Ecological Systems Theory
