# Supplementary material for: Psychological experiences of parents of adolescent patients with non-suicidal self-injury: a qualitative study based on Bronfenbrenner’s ecological systems theory
Source: BMC Psychiatry. 2025 Apr 11;25:366. doi: 10.1186/s12888-025-06812-5 (PMC11987188; doi:10.1186/s12888-025-06812-5)
Supplement: Supplementary file 2 — Additional file 2. Semi-structured interview guide. [file 12888_2025_6812_MOESM2_ESM.docx]

**Additional file**

**Semi-structured Interview Guide**

**Section 1**

The adolescent parents’ socio-demographic characteristics:

- Gender:
- Age:
- level of education:
- Marital status:
- The number of children:

**Section 2**

1. A brief introduction of the background and purpose of the study.

2. Notes for this interview, including audio recording, interview time, information confidentiality, etc.

3. Questions centered around gathering perceptions about the psychological experiences of parents of adolescent patients with non-suicidal self-injury (NSSI) based on Bronfenbrenner’s ecological systems theory. Can you tell me about this?

**Micro-system (home environment)**

- How do you feel when you first discover your child’s NSSI?
- What factors in the family do you think may contribute to NSSI in your child?
- What difficulties and challenges have you encountered in dealing with your child’s NSSI and how have you addressed them?

**Meso-system (interactions between the microsystems)**

- How has your child’s condition impacted your work and family?

**Exo-system (broader social environment)**

- Do you think the school and society have enough understanding and support for adolescents' NSSI behavior?
- What type of support do you currently need in this regard?

**Macro-system (social culture climate)**

- Does your social or cultural background have specific views or attitudes towards adolescents' NSSI behavior?

4. Thank you for your time. You will be invited to participate in the interview again for an intended future verification in our study.
